# Clinical efficacy analysis of Woodward's procedure for Sprengel deformity in children

**DOI:** 10.3389/fped.2026.1751083

**Published:** 2026-04-28

**Authors:** Tao Li, Wusheng Miao, Yinghan Lei, Hai Jiang

**Affiliations:** 1 Pediatric Orthopedics Department, Northwest Women’s and Children’s Hospital, Xi'an, China; 2 Pediatric Orthopedics Department, The Third Affiliated Hospital of Xi'an Medical University, Xi'an, China

**Keywords:** analysis, children, congenital shoulder deformity, Sprengel deformity, Woodward procedure

## Abstract

**Objective:**

Sprengel deformity is a rare congenital anomaly of the shoulder that leads to cosmetic and functional impairment. This study aimed to evaluate the clinical efficacy of the Woodward procedure in children, with a focus on both functional and cosmetic outcomes.

**Methods:**

A retrospective analysis was conducted on 13 pediatric patients with unilateral Sprengel deformity who underwent the Woodward procedure. The average follow-up was 3.9 years (range,1–12 years). Preoperative and postoperative assessments were performed using the Cavendish grading and Rigault classification systems. Shoulder abduction angles were measured, and associated anomalies were documented. Intraoperative “wake-up” test was performed to confirm brachial plexus integrity for severe cases.

**Results:**

The mean preoperative shoulder abduction was 114.2°. Postoperatively, significant improvements were observed in both cosmetic and functional outcomes. According to the Cavendish classification, 92.3% of patients achieved an excellent cosmetic result (grade 1). The Rigault classification showed 92.3% of patients improved to grade 1. The mean improvement was 2.1 grades for Cavendish and 1.5 grades for Rigault. For superior displacement, the mean value decreased substantially from 32.68 mm preoperatively to 11.33 mm postoperatively. Similarly, the mean rotational angle was reduced from 23.35° to 6.43° after surgery. Partial regeneration of the superior scapular angle was identified in two cases, with a residual omovertebral bone present in one. Complications were minimal, with no brachial plexus injuries or reoperations reported.

**Conclusion:**

The Woodward procedure proves to be a safe and effective surgical intervention for Sprengel deformity, providing significant and sustained improvements in both cosmetic appearance and shoulder function in pediatric patients.

## Introduction

Sprengel deformity represents the most common congenital anomaly of the shoulder girdle. This condition, characterized by the failure of the embryonic scapula to descend from its initial cervical position to its normal thoracic location by the end of the first trimester, presents a significant challenge in pediatric orthopedic practice. The resultant dysplastic and elevated scapula leads to a spectrum of functional and cosmetic concerns, including restricted shoulder abduction and flexion, scapular winging. An associated omovertebral bone that further tethers the scapula to the cervical spine is identified in more severe cases. Associated cervical or rib anomalies are commonly observed in these patients.

The primary goals of surgical intervention are to improve shoulder function, particularly abduction and forward elevation, and to restore a more natural shoulder contour, thereby minimizing the cosmetic deformity and its potential psychosocial impact. While several surgical techniques have been described, including the modified Green procedure (1–3), the classic or modified Woodward procedure has gained widespread acceptance as the preferred technique for many surgeons (4–8).

First described in 1961 (9), the Woodward procedure involves a midline approach to detach the paraspinal muscles (trapezius and rhomboids) from their spinous process origins, enabling direct and effective caudal relocation of the scapula. This technique offers distinct advantages, including better exposure for the excision of an omovertebral connection, a more robust soft-tissue repair by reattaching the muscles to a lower spinal level, and the potential for a more significant improvement in shoulder abduction. In this study, we will elaborate on the functional and cosmetic outcomes that can be anticipated in the management of this complex congenital condition.

## Methods

A retrospective review was conducted on 13 consecutive pediatric patients with Sprengel deformity who underwent the Woodward procedure at two different institutions. The study included patients who met the following criteria:

Inclusion Criteria: 1. Diagnosis of unilateral congenital high scapula. 2. Availability of standardized preoperative clinical photographs documenting cosmetic deformity and functional range of motion. 3. Availability of complete preoperative and postoperative imaging studies (e.g., radiographs and/or CT scans) and clinical data.

Exclusion Criteria: 1. Bilateral Sprengel deformity. 2. Absence of preoperative clinical photographs documenting appearance and function. 3. Incomplete preoperative or postoperative imaging or clinical records.

The majority of patients (10 out of 13, 76.9%) had associated congenital spinal anomalies. Spina bifida was the most common concomitant finding, present in 9 patients (69.2%). Other vertebral anomalies included scoliosis (2 patients) and hemivertebrae (2 patients). One patient also had a bifurcated rib. An omovertebral bone was identified and excised intraoperatively in 6 patients (46.2%). Preoperatively, all patients exhibited significant limitation in active shoulder abduction, with a mean preoperative abduction of 114.2° (range: 90° to 135°).

All patients were treated with the standard Woodward procedure, which included a midline incision, detachment of the trapezius and rhomboid muscles from their spinous process origins, inferior relocation of the scapula, and excision of any omovertebral bone when present. Functional outcome was primarily assessed by the improvement in active shoulder abduction angle, measured preoperatively and at the final follow-up.

After scapular relocation but before muscle reattachment, all long-acting agents are withheld, propofol/remifentanil stopped for 60–90 s until BIS ≥ 70 and TOF ≥ 0.9; the child is called to squeeze the examiner’s hand and flex the elbow—symmetrical voluntary power ≥ 3/5 confirms intact brachial plexus, any weakness prompts immediate release of traction or conversion to clavicular osteotomy, after which anaesthesia is promptly re-established.

The patients were followed for a mean duration of 3.9 years (range: 1 to 12 years) to monitor functional improvement and potential complications.

A paired samples t-test was employed to compare the preoperative and postoperative measurements. The *p*-value was determined to assess the statistical significance of the observed differences. A *p*-value of less than 0.05 (*p* < 0.05) was considered statistically significant.

## Results

According to the Cavendish grading system, the majority of patients (12 out of 13, 92.3%) had a severe preoperative deformity, classified as either grade 3 (10 patients) or grade 4 (3 patients). Postoperatively, the cosmetic outcome improved markedly. Twelve patients (92.3%) achieved an excellent cosmetic result (grade 1), and one patient (7.7%) had a good result (grade 2).

Similarly, evaluation using the Rigault classification, which radiologically assesses the height and rotation of the scapula, confirmed this improvement. Preoperatively, deformities were classified as grade 2 (9 patients) or grade 3 (4 patients). After surgery, 12 patients (92.3%) were downgraded to grade 1, indicating a normal or near-normal scapular position. The remaining patient improved to grade 2. The mean improvement was 2.1 grades for the Cavendish system and 1.5 grades for the Rigault system. Postoperatively, all patients exhibited significant improvement in active shoulder abduction, with a mean postoperative abduction of 167.3° (range: 150° to 180°).

**Figure 1:**
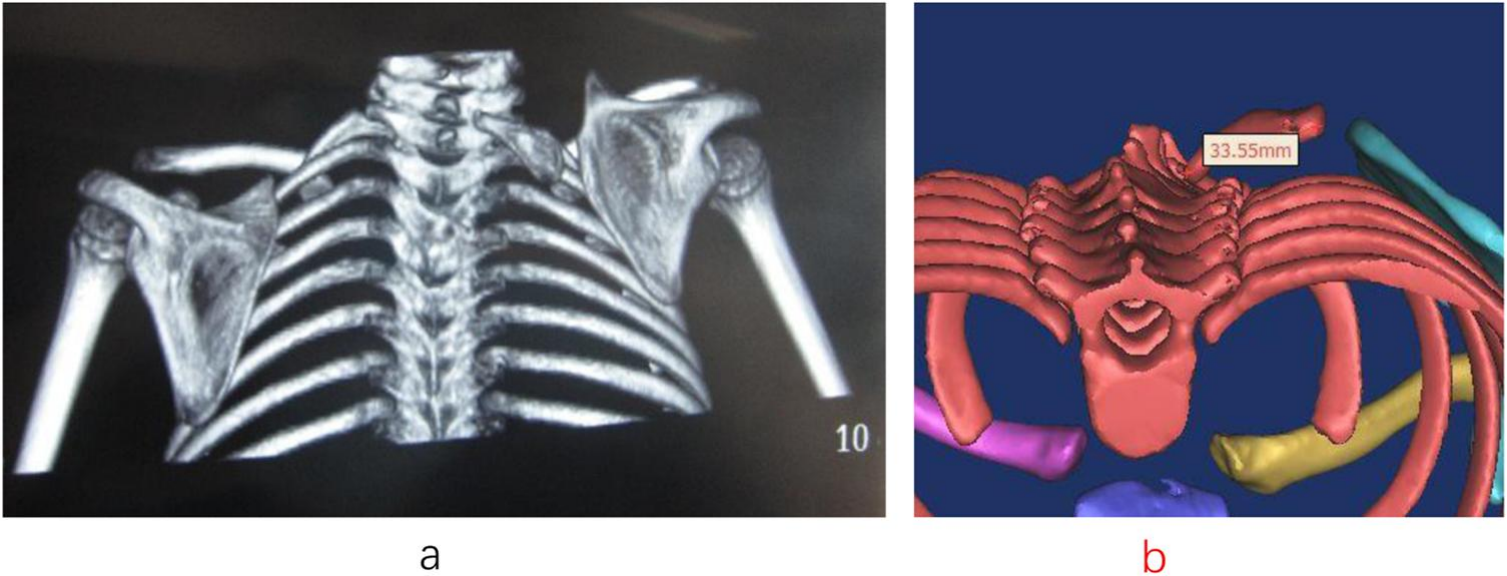
**(a, b)** Case 1: An omovertebral bone was found tethering the scapula to the cervical spine. The length was 33.55 mm of the omovertebral bone. The omovertebral bone was attached at the level of the C7 vertebra.

**Figure 2:**
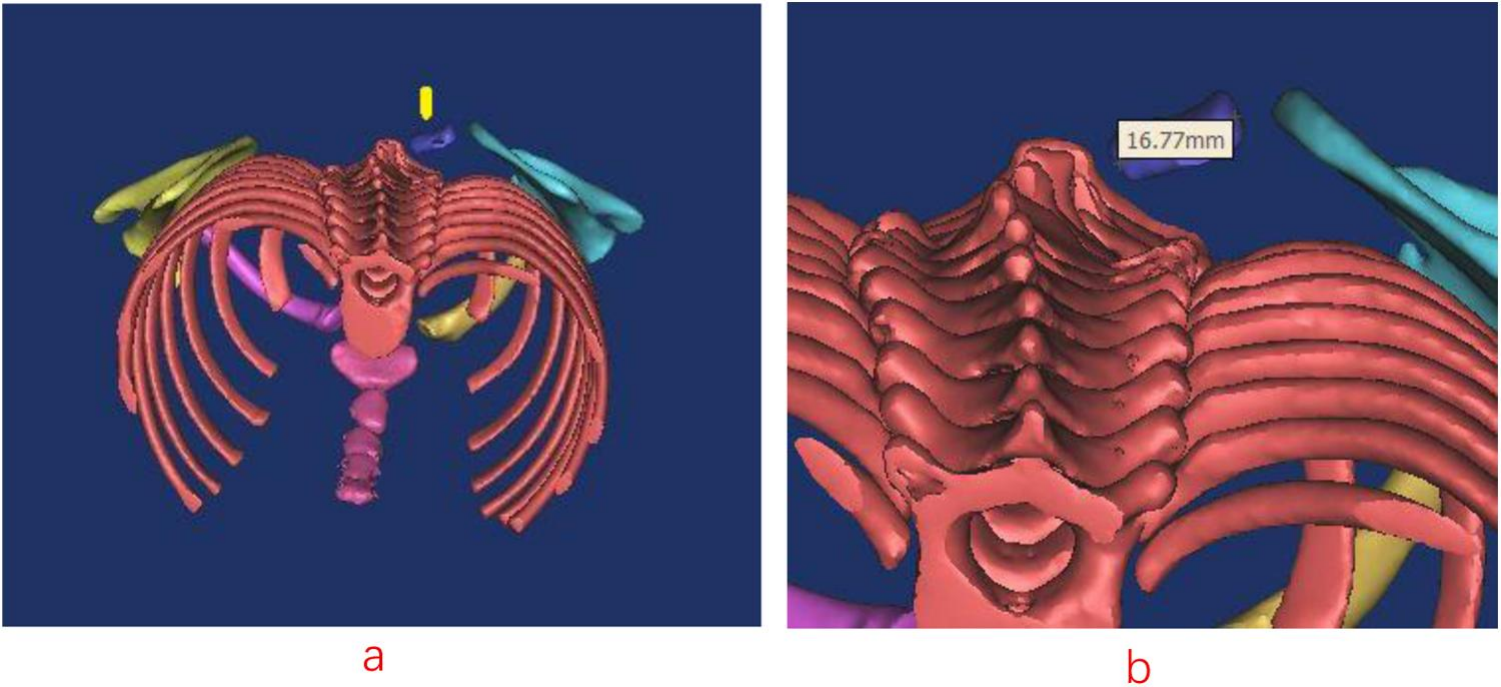
**(a, b)** Axial imaging demonstrates the residual omovertebral bone (yellow arrow) one week postoperatively. The length was 16.77 mm of the omovertebral bone.

**Figure 3:**
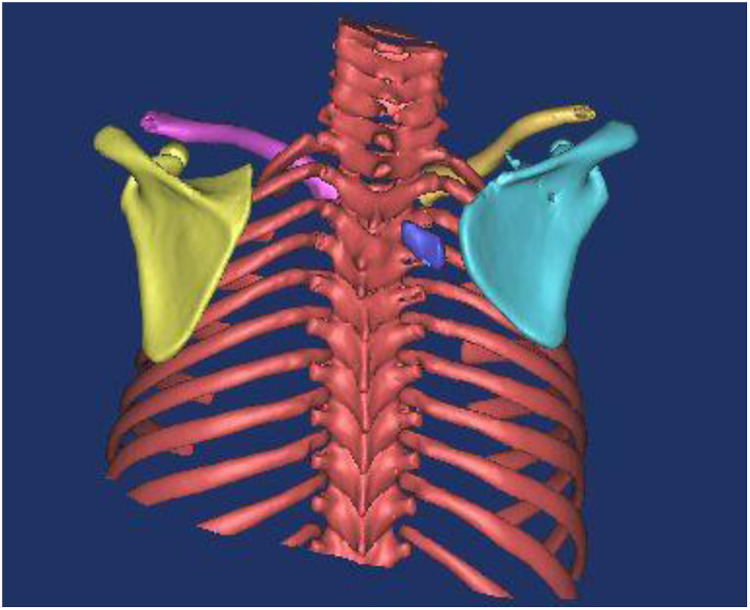
The postoperative anteroposterior (AP) radiograph demonstrates the resected superior angle of the scapula. The insertion point of the omovertebral bone was reduced to the T3 level.

**Figure 4:**
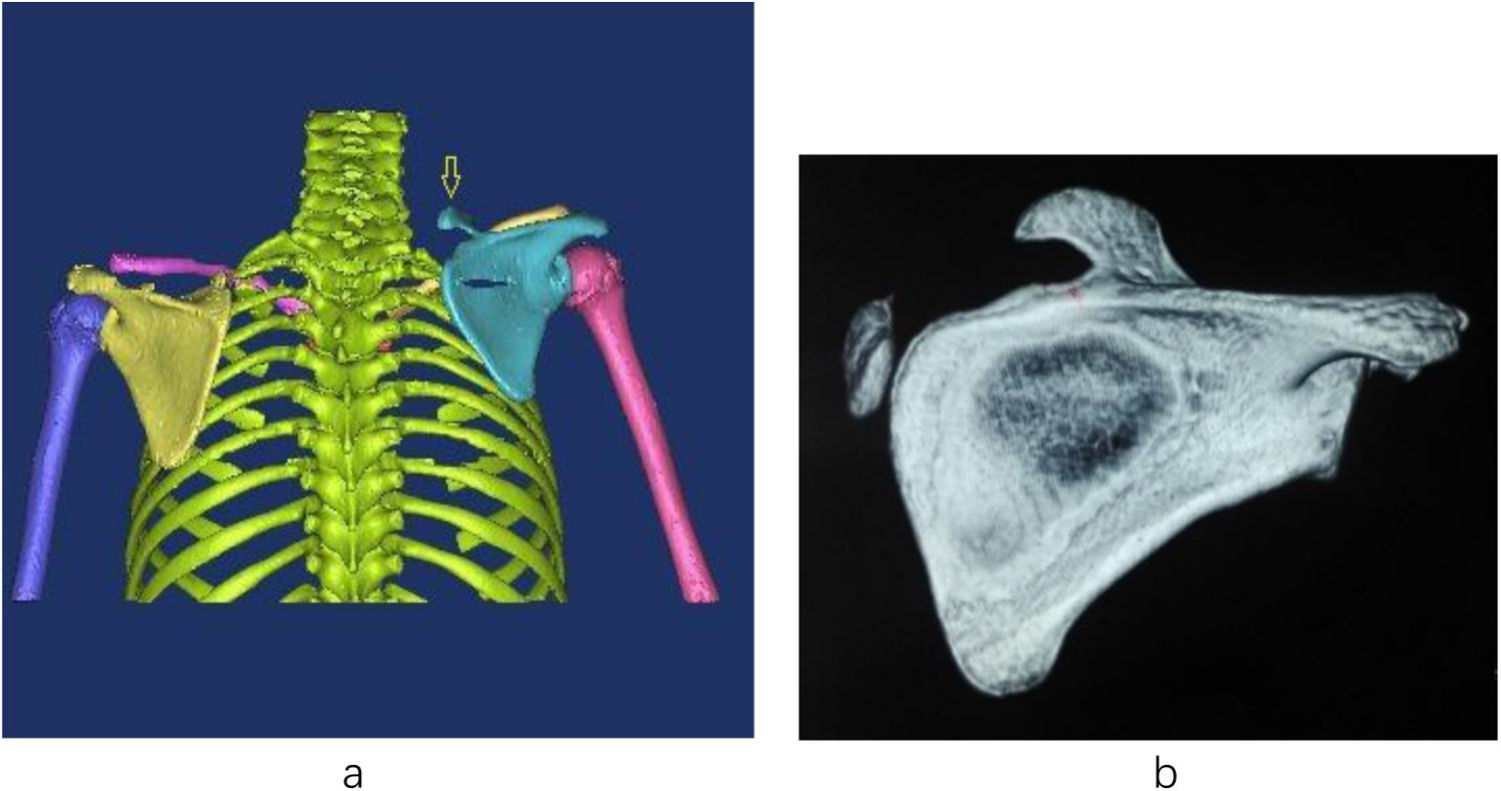
**(a, b)** At the 3-year follow-up, partial regeneration of the superior scapular angle was observed, and the residual omovertebral bone persisted.

**Figure 5:**
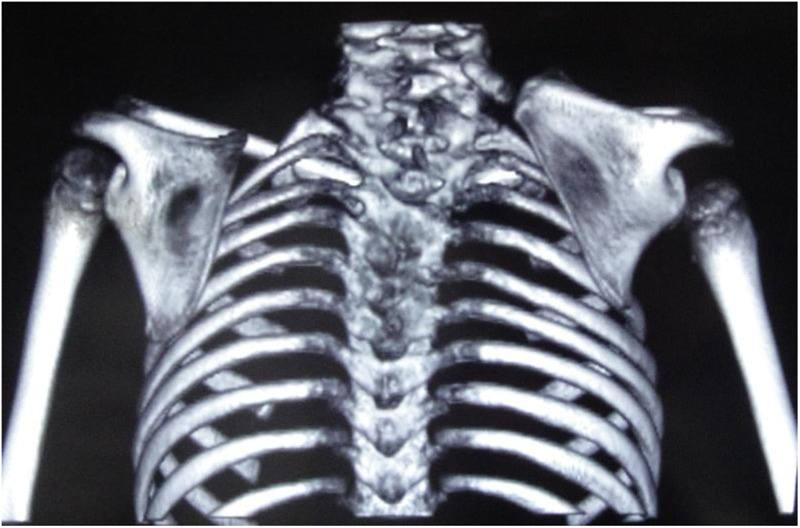
case 6. Preoperative 3D-CT demonstrated a significantly elevated right scapula and an omovertebral bone.

**Figure 6:**
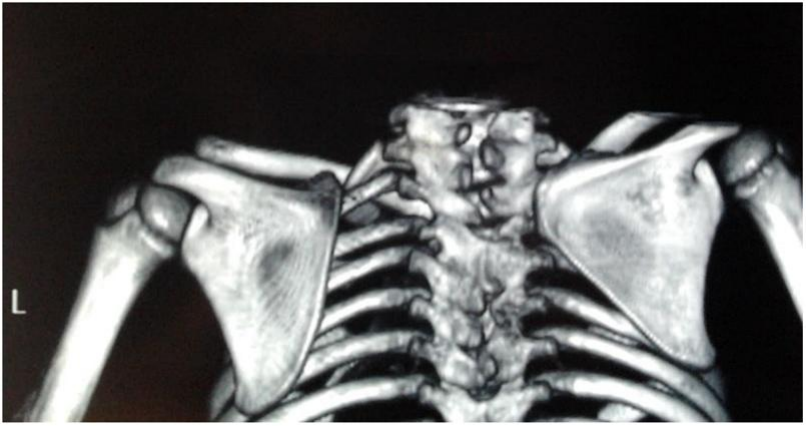
Postoperative 3D-CT confirmed successful descent of the right scapula to a level symmetric with the contralateral side, along with resection of the inferior angle.

**Figure 7:**
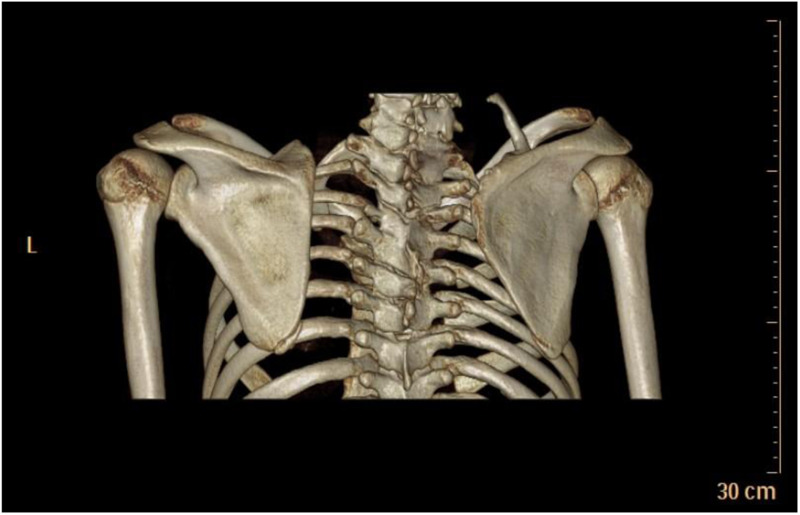
Follow-up imaging at 12 years revealed the formation of a columnar bone superior to the scapular border, originating from the regenerated inferior angle.

The omovertebral bone was completely resected in all but one case. In Case 1, a partial residual segment remained postoperatively (Figures 1, 2). Preoperatively, the omovertebral bone measured 33.55 mm in length. Following partial resection, its postoperative length was reduced to 16.77 mm. Anatomically, its insertion level shifted caudally from C7 to T3. At the 3-year follow-up, partial regeneration of the superior scapular angle was noted, and the residual omovertebral bone persisted (Figures 3, 4). In Case 6, after 12- year follow-up, the regenerated inferior angle formed a column of bone superior to the scapular border (Figures 5–7). The bone limited the rotational function of the cervical.

In 13 children with Sprengel deformity, both indices of scapular malposition fell markedly after the Woodward procedure. Superior displacement averaged 31.8 ± 12.5 mm pre-operatively and decreased to 10.7 ± 5.8 mm post-operatively, yielding a mean reduction of 21.1 mm (95% CI 15.3–26.9, *p* < 0.001, Cohen's d = 1.78). Likewise, the rotational angle diminished from 23.3 ± 9.9° to 6.3 ± 3.4°, an average improvement of 17.0° (95% CI 12.9–21.0, *p* < 0.001, Cohen's d = 1.95). These very large effect sizes confirm a statistically and clinically significant correction of scapular elevation and rotation.

Several complications occurred after surgery. In Case 5, a superficial skin infection was observed. The infection resolved with a two-week course of oral antibiotics. The only long-term complication was a curved scar in 2 patients. Over time, none of the patients required corrective procedures. No brachial plexus neuropathy was documented.

## Discussion

The presence of an omovertebral bone is a critical determinant in both the functional and aesthetic manifestations of Sprengel deformity. In our case series, 7 patients (over 50%) presented with an omovertebral bone. The rate was similar to other previous reports (10–12). This aberrant structure creates a rigid tether between the scapula and the axial skeleton, which directly restricts the glenohumeral joint's range of motion, particularly in abduction and flexion. This mechanical limitation leads to significant functional impairment in daily activities. Furthermore, the omovertebral bone contributes to the characteristic superior scapular elevation and rotation, exacerbating visible asymmetry, which is often a primary concern for patients and families. Therefore, surgical excision of this anomalous bone is a cornerstone of the corrective procedure. Its removal is essential not only to release the mechanical restraint and restore a functional arc of shoulder motion but also to facilitate the anatomical repositioning of the scapula, thereby addressing the core components of the deformity.

The significant improvement in active shoulder abduction—from a mean of 114.2° preoperatively to near-normal ranges postoperatively—highlights the procedure's ability to restore functional mobility. This is further supported by marked downgrading in both Cavendish and Rigault classifications, indicating substantial correction of scapular position and contour. The high percentage of patients achieving an excellent Cavendish grade (92.3%) postoperatively underscores the procedure's superior cosmetic results.

Long-term follow-up revealed rare but notable findings, such as residual or regenerated bone in two cases, which may affect cervical rotation or scapular movement over time. Releasing the spinal attachment of the omovertebral bone prevented the residual segment from restricting shoulder function and allowed it to settle into a more anatomic location. The phenomenon of bone regrowth warrants careful consideration. The most plausible etiology is incomplete resection of the cartilaginous anlage or periosteal bed of the omovertebral connection. In the pediatric population, retained osteoprogenitor cells possess a remarkable regenerative capacity, potentially leading to the formation of new bone. This regenerated bone, especially if it forms a rigid column, can tether the scapula and impair rotational function, as observed in our patient. Management of this complication should be guided by symptoms and functional deficit. Asymptomatic regrowth may be managed with observation. However, in cases presenting with pain, significant cosmetic concern, or functional impairment such as restricted cervical rotation or scapular movement, revision surgical excision is indicated. Preoperative 3D-CT imaging is crucial to delineate the precise anatomy of the regenerated bone and its relationship to surrounding neurovascular structures, facilitating safe and complete resection.

Minor complications, such as superficial infection and scar formation, were manageable and did not compromise overall outcomes. The absence of brachial plexus neuropathy in our series is particularly encouraging, reflecting the safety of the Woodward technique when performed with meticulous dissection and proper muscle reattachment.

In this study, an intraoperative wake-up test was employed to assess brachial plexus function in patients with severe deformity. As this test confirmed neural safety, clavicular osteotomy was avoided in all cases. These tests were performed in two patients with Cavendish grade 4 and two cases with superior displacement greater than 4 centimeters. This technique involves the temporary reversal of anesthesia, allowing for a direct clinical evaluation of active motor function in the upper limb following corrective maneuvers. Its principal advantage lies in providing real-time functional feedback, which is crucial for detecting nerve irritation or compression that may not be apparent on preoperative imaging or even with continuous electrophysiological monitoring. Successful execution of this test offers immediate and definitive confirmation of neural safety, enhancing the precision of correction and contributing to a significant reduction in the risk of iatrogenic neurological injury.

In previous studies, continuous intraoperative neuromonitoring (IONM) was utilized as a complementary strategy to mitigate the risk of brachial plexus injury (13–16). In contrast, modalities such as somatosensory evoked potentials (SSEPs) and especially motor evoked potentials (MEPs) deliver continuous information about the functional integrity of neural pathways throughout the critical phases of the operation. This allows for the immediate identification of a developing nerve compromise, often before it becomes irreversible. While IONM is highly sensitive, it can be susceptible to false-positive changes due to anesthetic or physiological variations and does not directly assess conscious motor command. In this series, intraoperative neuromonitoring was not utilized due to constraints involving required specialized equipment, the need for dedicated expertise, and concerns regarding potential false-positive results.

The use of clavicular osteotomy has been described in the literature as an established strategy to mitigate the risk of brachial plexus injury during the correction of severe Sprengel deformity (17–19). The procedure aims to reduce tension on neurovascular structures that may be exacerbated by the forceful caudal relocation of the scapula. By shortening the clavicular strut, the distance between the scapula and the cervical spine is effectively decreased, creating a more accommodating space for the brachial plexus and thus minimizing the risk of stretch-induced neuropraxia. As brachial plexus safety was confirmed by the intraoperative wake-up test, clavicular osteotomy was not performed in this series.

Despite the encouraging results, this study has several limitations that must be acknowledged. Firstly, the retrospective design inherits the potential for selection and information bias. The small sample size of 13 patients, while consistent with the rarity of the condition, limits the statistical power of our analysis and the generalizability of the findings. Furthermore, the follow-up duration, though a mean of 3.9 years, was highly variable (ranging from 1 to 12 years). This heterogeneity may not fully capture very long-term complications, such as the potential for late joint arthritis, scapular re-elevation, or the functional impact of bone regeneration as seen in one of our cases at the 12-year mark. The outcome assessment, while using validated classification systems, lacked a patient-reported functional outcome measure, which would have provided a more comprehensive view of the patients’ and parents’ satisfaction with daily activities and quality of life.

## Conclusions

In conclusion, the Woodward procedure offers a reliable and reproducible approach for correcting Sprengel's deformity. Its ability to achieve both functional and aesthetic improvements, with a low complication profile, supports its continued use as a first-line surgical treatment in children. Future prospective studies with larger, multi-center cohorts, standardized long-term follow-up, and the incorporation of patient-reported outcome measures are warranted to further validate these results and refine surgical indications and techniques.

## Data Availability

The datasets presented in this study can be found in online repositories. The names of the repository/repositories and accession number(s) can be found in the article/Supplementary Material.
