# Supplementary figures and images for: Clinical efficacy analysis of Woodward's procedure for Sprengel deformity in children
Source: Front Pediatr. 2026 Apr 28;14:1751083. doi: 10.3389/fped.2026.1751083 (PMC13161144; doi:10.3389/fped.2026.1751083)

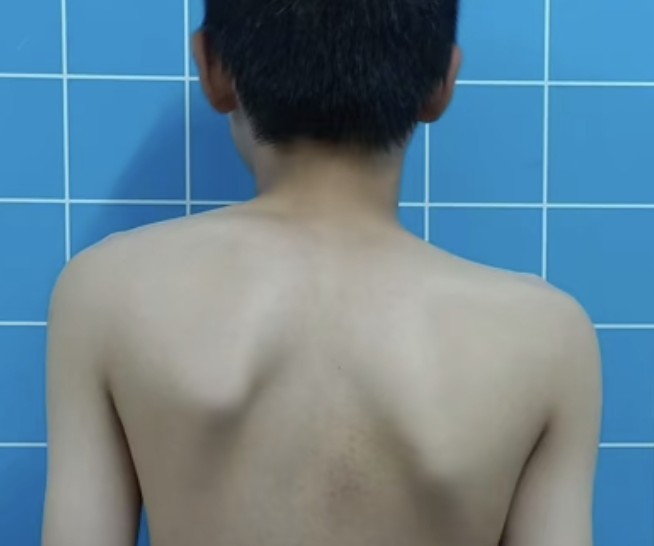

Supplement: Supplementary file 1 [file Image1.jpeg]

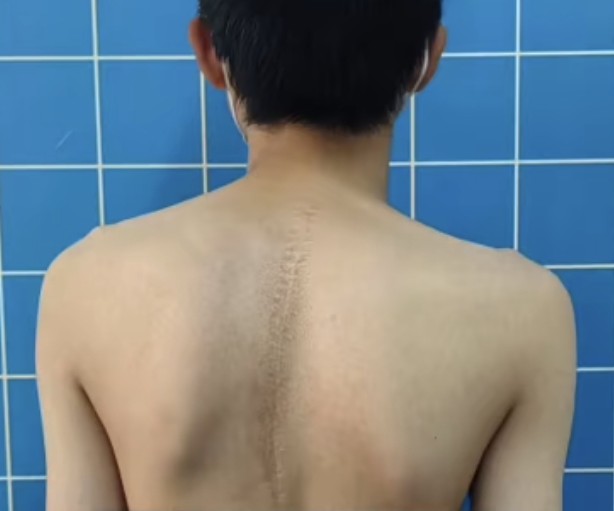

Supplement: Supplementary file 2 [file Image2.jpeg]

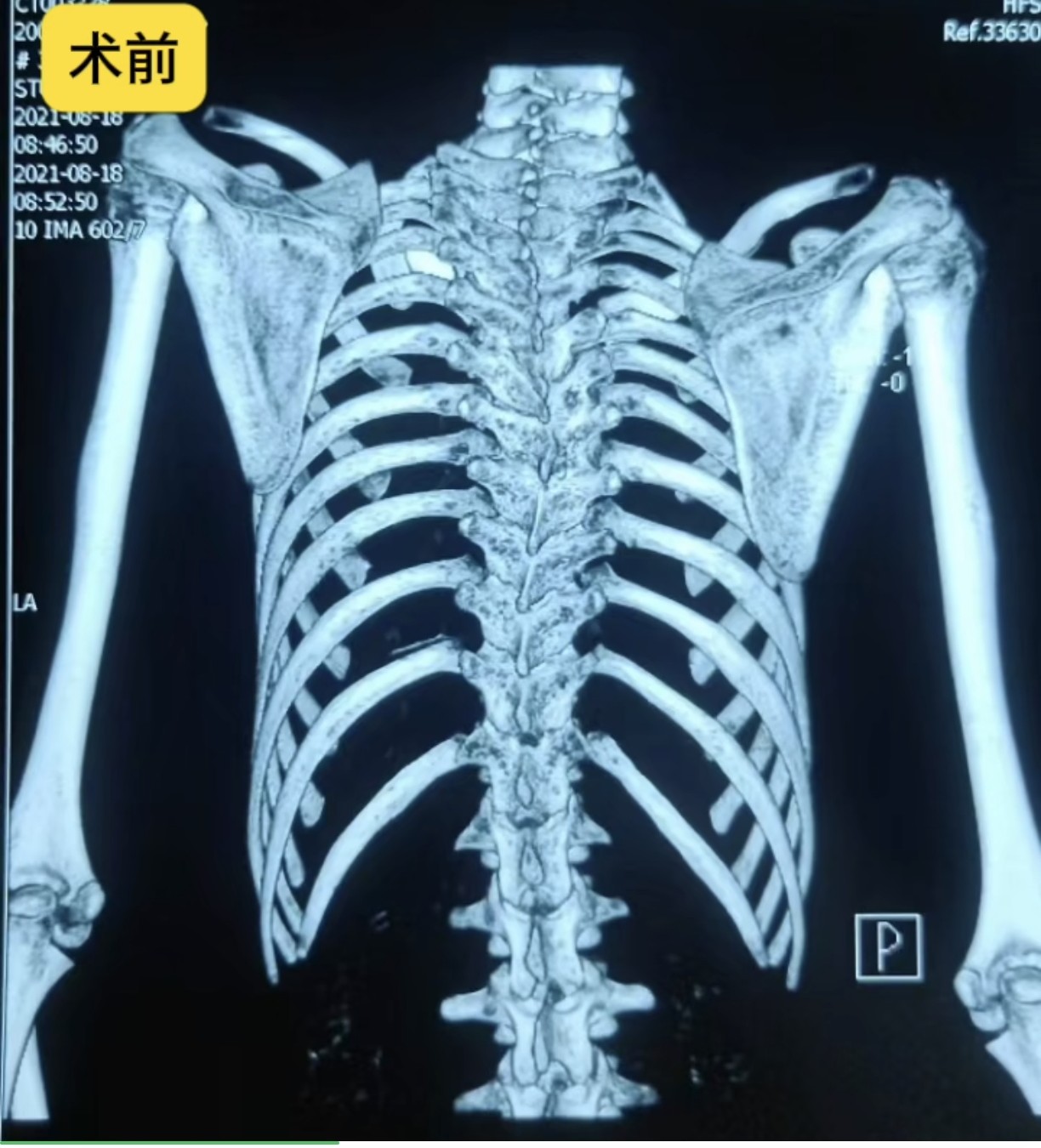

Supplement: Supplementary file 3 [file Image3.jpeg]

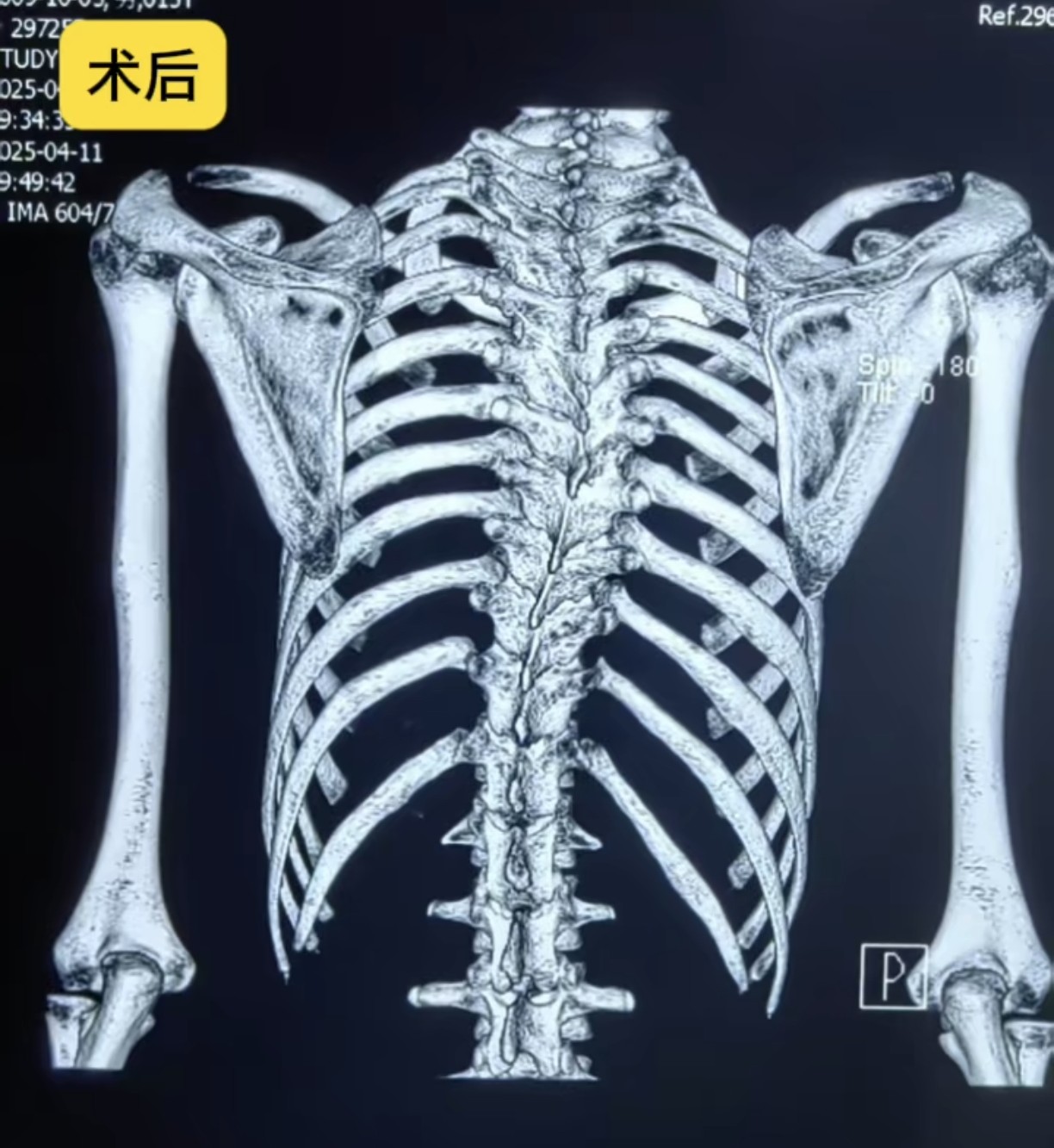

Supplement: Supplementary file 4 [file Image4.jpeg]

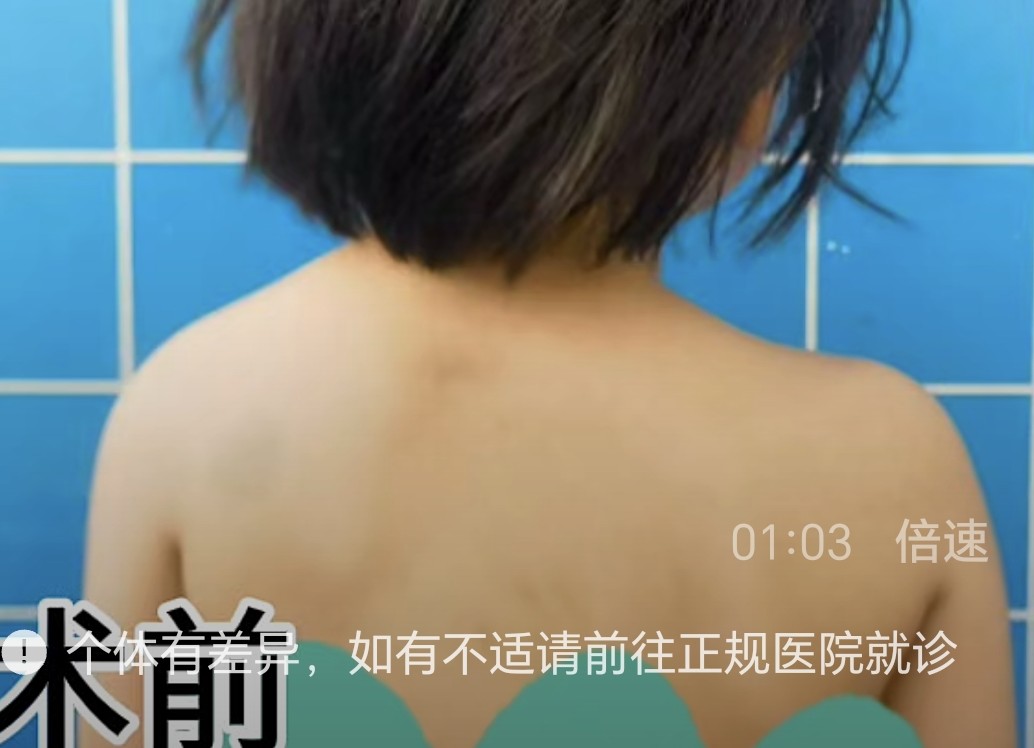

Supplement: Supplementary file 5 [file Image5.jpeg]

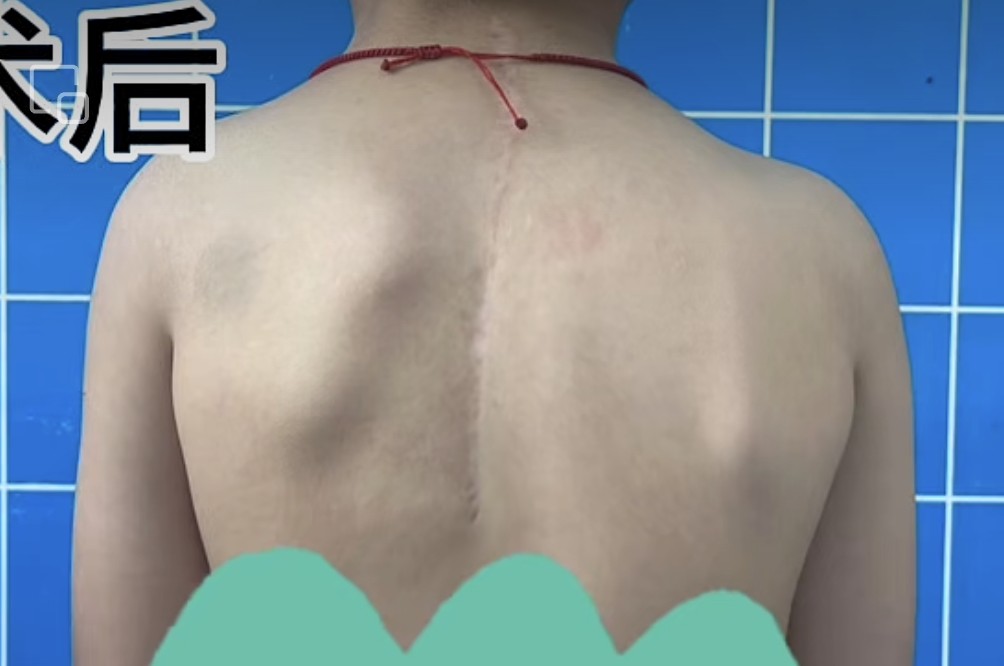

Supplement: Supplementary file 6 [file Image6.jpeg]

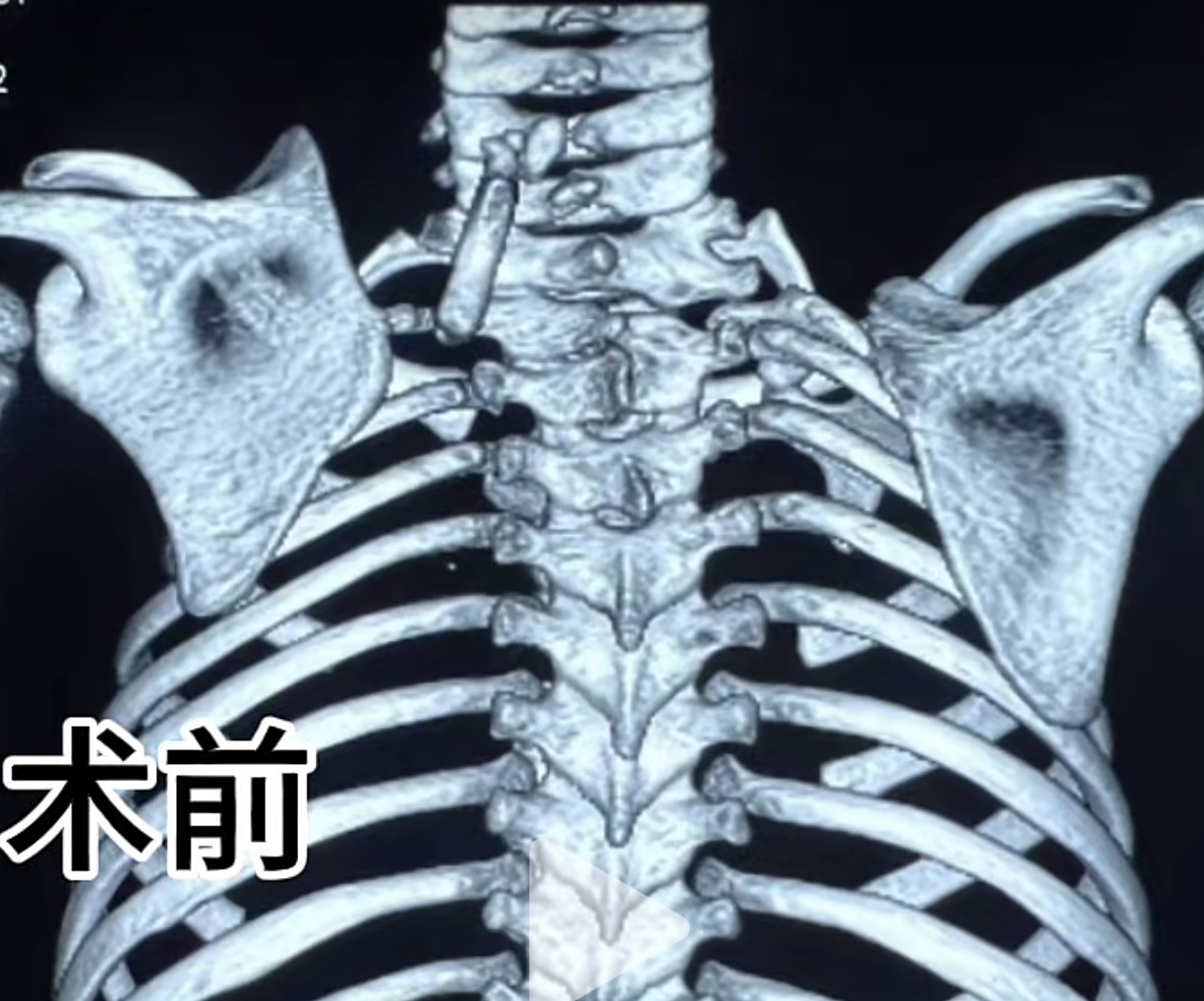

Supplement: Supplementary file 7 [file Image7.jpeg]

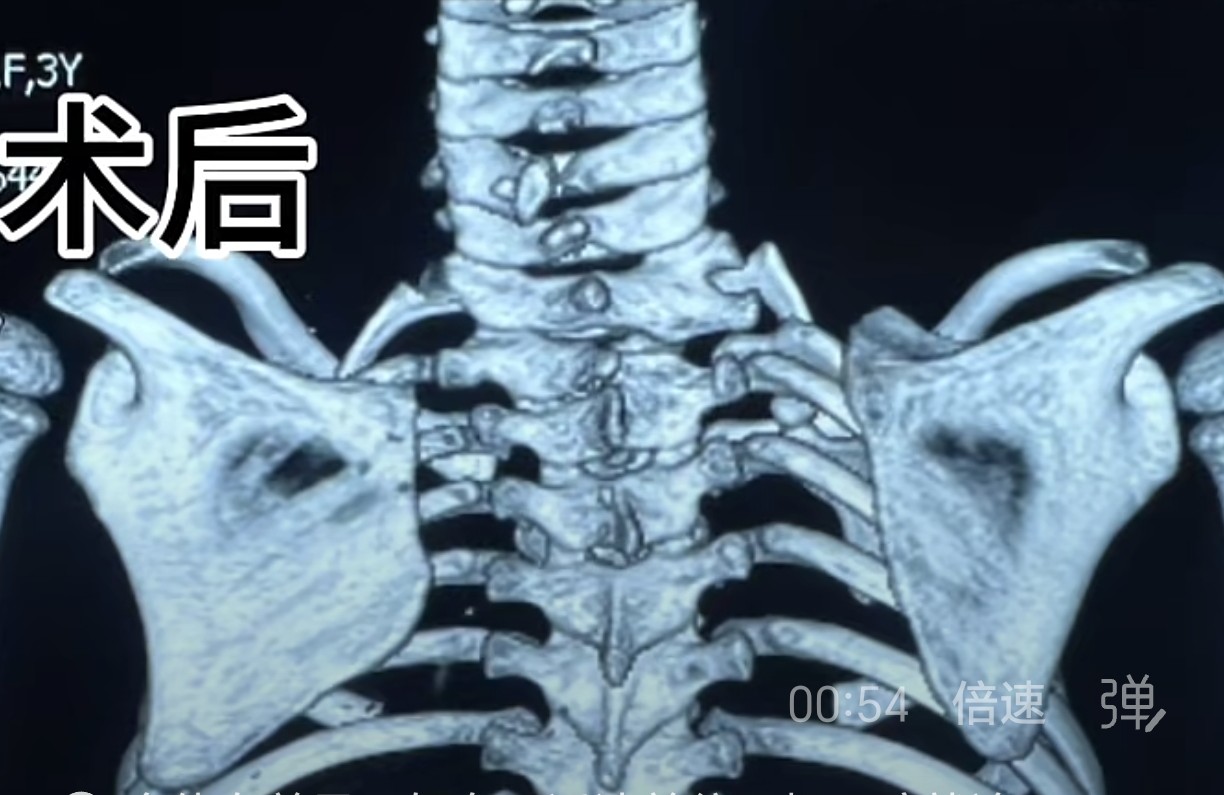

Supplement: Supplementary file 8 [file Image8.jpeg]
